# Naturally Occurring *Ehrlichia chaffeensis* Infection in Two Prosimian Primate Species: Ring-tailed Lemurs (*Lemur catta*) and Ruffed Lemurs (*Varecia variegata*)

**DOI:** 10.3201/eid0812.020085

**Published:** 2002-12

**Authors:** Cathy V. Williams, Jan L. Van Steenhouse, Julie M. Bradley, Susan I. Hancock, Barbara C. Hegarty, Edward B. Breitschwerdt

**Affiliations:** * Duke University Primate Center, Durham, North Carolina, USA; † Antech Diagnostics, Smyrna, Georgia, USA; ‡ North Carolina State University, Raleigh, North Carolina, USA

**Keywords:** *Ehrlichia chaffeensis*, ehrlichiosis, tick-borne diseases, primate, lemur

## Abstract

A naturally occurring infection of *Ehrlichia chaffeensis* in lemurs is described. DNA of *Ehrlichia chaffeensis* was identified by polymerase chain reaction in peripheral blood from six of eight clinically ill lemurs. Organisms were cultured from the blood of one lemur exhibiting clinical and hematologic abnormalities similar to those of humans infected with *E. chaffeensis.*

Phylogenetically, *Ehrlichia* species comprise an obligate intracellular group within the alpha *Proteobacteria* (1). A recent proposal by Dumler et al. resulted in reclassification of the members of the three *Ehrlichia* serogroups among the genera *Ehrlichia, Anaplasma*, and *Neorickettsia* (2). In addition to causing disease manifestations in humans and several domestic animal species, various *Ehrlichia* and *Anaplasma* species can be found in a wide range of wild animals that, in many instances, compose the blood reservoir from which ticks acquire infection for subsequent transmission to other mammals (3).

Nonhuman primates have been experimentally infected with *E. canis* or *E. equi* (recently reclassified as *A. phagocytophila*)(4–6), but, to our knowledge, natural infection of a nonhuman primate with an *Ehrlichia* or *Anaplasma* species has not been reported previously. We describe an epizootic of *E. chaffeensis* infection in a lemur colony, located in Durham, North Carolina.

## The Outbreak

Lemurs were housed at the Duke University Primate Center in two extended family groups of 9 and 10 animals, respectively. During the months of October 2000 through April 2001, lemurs were housed in wire-enclosed cages averaging 22 x 24 x 8 ft with access to indoor, heated rooms. On May 1, 2001, both groups were released into a 22.5-acre, fenced, mixed pine and deciduous hardwood forest in Durham for the summer.

From May 16 to June 25, 2001, anorexia, fever, lethargy, and lymphadenopathy developed in seven ring-tailed lemurs (*Lemur catta*) and one red ruffed lemur (*Varecia variegata rubra*), ranging in age from 14 months to 17 years. *Amblyomma americanum*, *Rhipicephalus sanguineus,* and *Dermacentor variabilis* adult ticks were found on lemurs at the time of illness, but the numbers of ticks were not quantified.

At the onset of illness, animals received a physical exam at which time blood was drawn for complete blood counts and serum chemistry profiles. Additional EDTA-anticoagulated peripheral blood was stored at –80°C for subsequent DNA isolation and polymerase chain reaction (PCR) amplification. Two milliliters of EDTA-anticoagulated blood were maintained at room temperature for attempted isolation of *Ehrlichia* organisms. Urine was collected either as a voided midstream sample or by cystocentesis. Specimens collected from inguinal lymph nodes by fine needle aspiration were submitted to a commercial laboratory for evaluation by a cytopathologist. Peripheral blood smears and lymph node aspirates were stained with a Wrights-Giemsa stain and evaluated by light microscopy.

Because of the lack of published comprehensive normal blood values for ring-tailed lemurs, complete blood counts and serum chemistry profile results obtained from 18 clinically healthy ring-tailed lemurs that had undergone routine physical examinations at the Duke University Primate Center during the years 1995 through 2000 were used for comparison to values obtained from the *E. chaffeensis–*infected lemurs. As normal clinical pathology values for red ruffed lemurs vary minimally from that of normal ring-tailed lemurs described here, data from the eight ill animals were analyzed as a single group rather than as separate species (7).

Thrombocytopenia was the most commonly observed hematologic abnormality, followed by lymphopenia, leukopenia, and neutropenia. Hematocrit values were normal to elevated in all animals (Table). Hyperbilirubinemia in the presence of low to normal serum alkaline phosphatase values was the most common biochemical abnormality, followed by azotemia (increased urea nitrogen and creatinine), hyponatremia, hypochloremia, hyperglycemia, mild increases in serum transferase activities, and hypoproteinemia with corresponding hypoalbuminemia. Proteinuria (urine dipstick values ranging from 2+ to 3+) was detected in five of seven lemurs in which urinalysis was performed. Protein/creatinine ratios ranged from 0.4 to 1.2. Urine protein/creatinine ratios in lemurs without detectable protein by urine dipstick examination ranged from 0.1 to 0.2 (data not shown). Morulae were seen in lymphocytes and monocytes in lymph node aspirates from three of eight clinically ill lemurs (Figure). Morulae were not seen in leukocytes in peripheral blood smears, although examinations of buffy coat smears were not performed.

**Table:** Comparison of blood parameters from normal and *Ehrlichia chaffeensis–*infected lemurs^a^

	Normal (n=18)			Infected (n=8)		
Test	Mean	SD^b^	Range	Mean	SD	Range
Glucose (mg/dL)	127	55	55–238	226	88	96–337
Urea nitrogen (mg/dL)	20	5	8–29	38	14	15–57
Creatinine (mg/dL)	0.9	0.1	0.7–1.2	1.7	0.2	1.2–1.9
Total protein (g/dL)	7.8	0.3	7.1–8.2	7.0	0.9	5.3–8.1
Albumin (g/L)	5.7	0.3	5.2–6.6	5.0	0.7	3.8–6.0
Total bilirubin (mg/dL)	0.4	0.1	0.3–0.6	1.0	0.2	0.6–1.3
Alkaline phosphatase (IU/L)	240	83	125–476	89	38	44–144
Aspartate transferase (IU/L)	46	26	20–128	86	41	33–155
Alanine transferase (IU/L)^c^	81	51	10–210	128	85	47–321
Sodium (mg/dL)	146	4	141–152	137	3	134–142
Chloride (mg/dL)	104	4	97–111	97	4	91–103
Platelets (x10^3^/μL)	327	132	165–685	181	135	34–410
Packed cell volume (%)	51.1	3.6	45.6–57.6	55.1	5.9	46–62.4
WBC (x10^3^/μL)^d^	6.1	1.5	3.9–8.8	5.0	2.9	1.0–9.5
Neutrophils (x10^3^/μL)^d^	3.6	1.3	0.3–6.0	2.8	1.9	0.3–6.3
Lymphocytes (x10^3^/μL)	2.7	1.5	1.1–5.4	1.6	1.0	0.2–3.0

^a^Variation between means of normal and infected animals is significant at a confidence level of p<0.05 using the 2-sample t test, except where otherwise specified. ^b^SD, standard deviation. ^c^p=0.18. ^d^p=0.34; WBC, leukocyte count.

**Figure:**
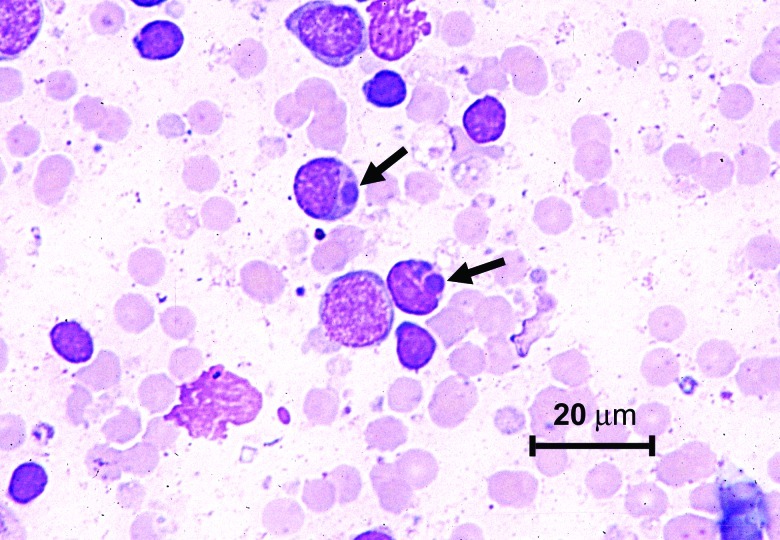
Photomicrograph of a lymphocyte from a lymph node aspirate containing *Ehrlichia* morula (arrow). Stain is with Wright’s-Giemsa.

DNA extraction was performed with commercially available QIAmp Blood kit (Qiagen, Chatsworth, CA) from 200 μL EDTA blood samples that had been frozen at –80°C. PCR was performed in a two-step method as previously described (8), first using primers specific for the genera *Ehrlichia* and *Anaplasma* and then using primer pairs specific for *E. canis, E. chaffeensis, E. ewingii,* and *A. phagocytophila,* on all genus-positive samples. Culture-grown *E. chaffeensis* was used as a positive control. *E. chaffeensis* DNA was amplified from blood samples from six of eight affected animals. Coinfections with multiple *Ehrlichia* spp. were not found.

Leukocytes obtained from a clinically ill red ruffed lemur were isolated and cultured in DH82 cells at 37°C with 5% CO_2_ as described (9). After 16 days in culture, many morula-like inclusions were visible on stained cytospin preparations. A postinoculation day 16 sample of the cultured cells was also processed for PCR amplification. Primers specific for *E. chaffeensis* yielded an amplicon of the appropriate size.

The clinical condition of animals improved rapidly after treatment with doxycycline, 5 mg/kg orally twice daily, was initiated. Improvement in the degree of fever and anorexia were noted as early as 24 hrs after the start of treatment in all animals. Time to complete resolution of clinical symptoms varied depending on the severity of clinical signs, but all lemurs appeared behaviorally normal by day 7 of therapy. Similarly, hematologic values were normal for the seven lemurs in which laboratory tests were repeated 12–87 days after doxycycline therapy was completed.

## Conclusions

An unanticipated series of events created the opportunity for an epizootic of *E. chaffeensis* infection involving lemurs, as described in this report. In association with fence maintenance and construction during the winter, the inadvertent introduction of several white-tailed deer into the lemur’s 22-acre summer enclosure facilitated the transport of ticks onto the facility. In addition to *A. americanum*, *R. sanguineus* and *D. variabilis* ticks were removed from the lemurs at the time of illness. *R. sanguineus*, the brown dog tick, most often feeds selectively on dogs and is the vector for *E. canis* infection. *D. variabilis*, the common dog tick, is known to feed on deer as well as many other small to medium mammals and is most commonly associated with Rocky Mountain spotted fever. *Amblyomma americanum* is considered the most important vector tick for transmission of *E. chaffeensis,* and deer are an important wildlife reservoir for *E. chaffeensis* in nature (10,11). Considered a very aggressive tick species, it will feed on numerous wild and domestic animals, as well as humans. In conjunction with the substantial increase in the deer population in the southeastern United States, human and animal exposure to *A. americanum* has increased dramatically, coincident with a gradual northern expansion of the range of this tick species, particularly in the eastern and central United States (1,10). As a result, the incidence of human ehrlichiosis has increased dramatically (12). Similar to *Rickettsia rickettsii*, the cause of Rocky Mountain spotted fever, *E. chaffeensis* causes substantial illness and annual case-fatality rates that range from 5% to 10% of reported human cases (1).

The inadvertent exposure of lemurs to *E. chaffeensis* resulted in clinical and hematologic abnormalities comparable to those reported in dogs (8,2) and humans; these abnormalities included thrombocytopenia, neutropenia, leukopenia, and lymphopenia (13,14). Similar to humans, *E. chaffeensis–*infected lemurs developed mild hyperbilirubinemia and increases in serum aminotransferase levels. Similar to dogs experimentally infected with *E. canis*, lemurs developed proteinuria, which resolved after therapeutic elimination of *E. chaffeensis* (15). Despite anorexia and accompanying dehydration, which should result in hypernatremia and hyperchloremia, serum sodium and chloride values were below the reference range in six of seven ill lemurs. Although reported in individual cases (14), hyponatremia and hypochloremia are not typically associated with human *E. chaffeensis* infections (12,13). Hyperglycemia in the affected animals was attributed to stress associated with restraint in connection with the concurrent illness.

Previous reports of experimental infections of nonhuman primates with *Ehrlichia* or *Anaplasma* suggest that susceptibility may vary with the species of primate infected as well as the infective agent**.** In 1936, Donatien and Lestoquard reported severe disease following experimental infection of long-tailed macaques (*Macaca fasicularis)* with *E. canis,* whereas infection of vervet monkeys (*Cercopithecus pygerythrus*) did not induce disease (5). Neutrophilic morulae, fever, and anemia were observed in rhesus macaques (*Macaca mulatta)* and baboons (*Papio anubis*) experimentally infected with *A. phagocytophila,* but behavioral abnormalities were not observed (6). In a more recent study, two rhesus macaques experimentally infected with *A. phagocytophila* developed pyrexia, lethargy, neutropenia, thrombocytopenia, anemia, and morulae in monocytes and neutrophils (7). Collectively, these reports indicate that disease manifestations can develop in nonhuman primates when they are infected with *E. canis, A. phagocytophila,* or *E. chaffeensis.*

In summary, after transmission of *E. chaffeensis,* presumably by *A. americanum*, disease manifestations very similar to those reported in human patients developed in lemurs in a research colony. For diagnosis, morula may be found in monocytes and lymphocytes in lymph node aspirates. *E.* *chaffeensis* can be isolated in tissue culture and *E. chaffeensis* DNA can be amplified from peripheral EDTA anti-coagulated blood samples. Treatment with doxycycline, the drug of choice for treating human and canine ehrlichiosis, elicits a dramatic clinical and hematologic response. The impact of vector-borne diseases should be considered when working with nonhuman primate colonies maintained in natural environments in tick-endemic areas.^1^

## Footnotes

1 Approximately 1 year after the outbreak in this report, *E. chaffeensis* was amplified by PCR from an ill lemur in Virginia that was unrelated to the Duke colony. The clinical and hematologic manifestations were consistent with those seen in lemurs in this report.
